# A smartphone- and wearable-based biomarker for the estimation of unipolar depression severity

**DOI:** 10.1038/s41598-023-46075-2

**Published:** 2023-11-01

**Authors:** Ahnjili Zhuparris, Ghobad Maleki, Liesbeth van Londen, Ingrid Koopmans, Vincent Aalten, Iris E. Yocarini, Vasileios Exadaktylos, Albert van Hemert, Adam Cohen, Pim Gal, Robert-Jan Doll, Geert Jan Groeneveld, Gabriël Jacobs, Wessel Kraaij

**Affiliations:** 1 Centre for Human Drug Research (CHDR), Zernikedreef 8, 2333CL Leiden, The Netherlands; 2 Leiden University Medical Centre (LUMC), Leiden University, Leiden, The Netherlands; 3 Department of Psychiatry, Leiden University Medical Center (LUMC), Leiden, The Netherlands; 4 Leiden Institute of Advanced Computer Science (LIACS), Leiden University, Leiden, The Netherlands; 5 Transparant Centre for Mental Health Care, Leiden, The Netherlands

**Keywords:** Diagnostic markers, Predictive markers, Prognostic markers, Biomarkers, Outcomes research, Clinical trial design, Translational research

## Abstract

Drug development for mood disorders can greatly benefit from the development of robust, reliable, and objective biomarkers. The incorporation of smartphones and wearable devices in clinical trials provide a unique opportunity to monitor behavior in a non-invasive manner. The objective of this study is to identify the correlations between remotely monitored self-reported assessments and objectively measured activities with depression severity assessments often applied in clinical trials. 30 unipolar depressed patients and 29 age- and gender-matched healthy controls were enrolled in this study. Each participant’s daily physiological, physical, and social activity were monitored using a smartphone-based application (CHDR MORE™) for 3 weeks continuously. Self-reported depression anxiety stress scale-21 (DASS-21) and positive and negative affect schedule (PANAS) were administered via smartphone weekly and daily respectively. The structured interview guide for the Hamilton depression scale and inventory of depressive symptomatology–clinical rated (SIGHD-IDSC) was administered in-clinic weekly. Nested cross-validated linear mixed-effects models were used to identify the correlation between the CHDR MORE™ features with the weekly in-clinic SIGHD-IDSC scores. The SIGHD-IDSC regression model demonstrated an explained variance (R^2^) of 0.80, and a Root Mean Square Error (RMSE) of ± 15 points. The SIGHD-IDSC total scores were positively correlated with the DASS and mean steps-per-minute, and negatively correlated with the travel duration. Unobtrusive, remotely monitored behavior and self-reported outcomes are correlated with depression severity. While these features cannot replace the SIGHD-IDSC for estimating depression severity, it can serve as a complementary approach for assessing depression and drug effects outside the clinic.

## Introduction

An ideal biomarker would serve as a dynamic indicator of disease activity. The biomarker should be capable of predicting changes in disease progression over time, regardless of the treatment intervention^1,2^. By leveraging advanced machine learning algorithms, researchers can integrate multiple objective biomarkers into composite biomarkers, enabling a more comprehensive and multifaceted understanding of disease activity and the impact of treatment interventions. Drug development for the treatment of depression is expected to benefit greatly from robust biomarkers that reflect the etiology, phenomenology, and treatment management of the disorder. Depression is not only associated with subjective symptoms such as sadness, despair, and anhedonia, but also with negative behavioral and neurovegetative effects such as decreased psychomotor activity and changes in appetite and sleep. A combination of objective physiological indicators and frequent subjective assessments can potentially be used as features to create a composite biomarker to estimate the presence or severity of depression, or even to quantify the effects of therapeutic interventions with drugs and/or psychotherapy.

The current gold standards for assessing depression severity and treatment effects, such as the Hamilton depression rating scale (HAMD) and the Montgomery & Åsberg depression rating scales (MADRS), are clinician-administered questionnaires^3,4^. As these questionnaires require an interview with a clinician, they are applied infrequently, and thus real-time behavioral assessments of depressed individuals cannot be obtained^5^. Further, retrospective self-reported appraisals can be compromised by recall bias and altered by socially desired reporting from patients^6,7^. By relying on the current gold standards for the assessment of depression severity, researchers routinely miss out on real-time and real-world behavioral patterns associated with depression, which may potentially attenuate treatment effects. To address such limitations, there is a demand for developing and validating methodologically sound biomarkers to quantify depression severity in real-time under free-living conditions.

Mobile health (mHealth) biomarkers are biomarkers derived from mobile health technologies, such as smartphones, wearables, and other portable devices that can be worn outside a controlled setting^8^. Emerging literature on depression and mHealth biomarkers supports the notion that smartphones and wearable devices can overcome the limitations of traditional depression rating scales. The sensors embedded in these devices (e.g. accelerometers, global positioning systems (GPS), and microphones) provide real-time, unobtrusive, passively collected data relating to behavioral patterns exhibited under free-living conditions^9–13^. In turn, these data can offer insights into an individual’s sleep rhythms^14^, social interactions^15^, and daily physical activities^16^, all of which can be useful for quantifying depression severity. While the existing body of evidence demonstrates that these digital mHealth biomarkers can be used to identify the presence of depressive symptoms or the estimation of daily mood, however, there are still three major critical gaps that remain to be understood. First, several studies in this field have relied on self-reported psychometric assessments, such as the depression anxiety and stress scale (DASS), the Positive and Negative Affect Schedule (PANAS), and quick inventory of depressive symptomatology (QIDS), for documenting depression severity^17,18^. To date, we have only identified two studies that correlated digital mHealth biomarkers sourced from smartphone and wearable data with clinician’s assessment of depression among unipolar depressed patients^19,20^. Therefore, more evidence is required for corroborating the clinical validity of these remotely monitored biomarkers in depression clinical trials. Next, these studies rarely include age- or sex-matched non-depressed controls. Healthy controls can also present behaviors and symptoms observed among depressed patients^21^. Observing behaviors exhibited by both depressed and non-depressed controls enables the identification of behaviors specific to depressed patients. This allows for the discovery of new candidate drugs that target the core symptoms of unipolar depression. Lastly, determination of the optimal monitoring period and data resolution needed for developing depression biomarkers has been overlooked in previous studies. Depression is a highly variable and heterogenous disorder^22^; thus, an effective depression biomarker should consistently correspond with the heterogenous changes in depression over time. While the advances of remote sensing can provide researchers with fine-grain longitudinal datasets, it can be operationally and financially burdensome for patients and researchers to collect, store, and process such expansive and information-dense datasets. Therefore, evaluating how much data is required to identify the earliest, reliable, and minimally observable changes in the patients’ clinical status is crucial. This evaluation is necessary to minimizing the impact of data collection on both the patients and researchers.

The current study consisted of two research objectives. First, we investigated the correlation of clinical ratings of depression, among unipolar depressed patients and healthy controls, with remotely self-reported psychometric assessments and smartphone- and wearable-based features. Here, we defined features as individual measurable variables, such as average heart rate or total steps. Second, we examined how many data points are required to develop a reliable statistical model that can consistently estimate the longitudinal variability of depression. The primary objective allows for the identification of reliable and clinically relevant depression biomarkers that can be monitored continuously in real-world conditions. The secondary objective focuses on the validation of a minimum dataset required to maintain the accuracy, sensitivity, and specificity of the biomarkers. To achieve these objectives, we adopted linear mixed effects models to estimate the weekly structured interview guide for the Hamilton depression scale and inventory of depressive symptomatology (SIGHD-IDSC) clinician ratings using 1, 2, and 3 weeks of remotely collected data. Together, such correlated features can potentially represent a composite digital mHealth biomarker for monitoring depression severity in longitudinal clinical trials.

## Methods

### Study overview

This was a cross-sectional, non-interventional pilot study conducted by Centre for Human Drug Research (CHDR) and Transparant Centre for Mental Health in Leiden, The Netherlands. The participants were monitored between March 2019 and March 2020. Prior to any assessments, patients provided written informed consent. The trial was approved by the Stichting Beoordeling Ethiek Biomedisch Onderzoek ethics committee, Assen, the Netherlands, and was conducted in accordance with the Declaration of Helsinki at the Centre for Human Drug Research, Leiden, The Netherlands.

### Participants

Eligible patients and healthy controls were between the ages of 18–65 years old and had a body mass index (BMI) between 18 and 30 kg/m^2^. Patients and healthy controls with severe coexisting illnesses that might interfere with study adherence or pregnant were excluded. Patients and healthy controls were required to use their own Android smartphone (version 5.0 or higher) as the CHDR MORE™ app was only available on Android App Store. Due to the Apple operating systems restrictions, the iPhone user device logs could not be accessed by the app.

Eligible patients had either a diagnosis of Major Depressive Disorder (MDD) without psychotic features or Persistent Depressive Disorder (PDD) according to the DSM-IV (Diagnostic and Statistical Manual of Mental Disorders) or DSM-V. The diagnosis was provided by an attending general practitioner, psychologist, or psychiatrist and was confirmed with the Mini International Neuropsychiatric Interview (MINI) version 7.0. To be included in the study, each patient must have had a structured interview version of Montgomery-Åsberg depression rating scale (MADRS-SIGMA) score of more than 22 at screening. Further, the patients either received no antidepressant drug treatment at least 2 weeks prior to screening, or they were receiving an antidepressant drug treatment with a stable dose for at least 4 weeks prior to screening. Patients were excluded if they presented specific psychiatric co-morbidities (psychotic disorder, bipolar disorder, mental retardation, or cluster B personality disorders), presented a Columbia-suicide severity rating scale (C-SSRS) greater than 5, alterations of antidepressant drug (including dose) during the trial period or use of sedative medications within 2 weeks of the beginning of the clinical trial. This was confirmed by their general practitioner, psychologist, or psychiatrist.

Eligible healthy controls were included if they had no previous or current history (or family history) of psychiatric disorder or chronic co-morbidities. Healthy controls were age and sex-matched with the MDD and PDD patients.

Participants received monetary compensation for their time and effort. The reimbursement was determined by a schedule approved by the Ethics Committee and was based on the amount of time the participants had to spend participating in the study. This compensation was not linked to the quantity or quality of the data obtained.

### CHDR MORE™ and withings devices

On Day 0 of the trial, the CHDR MORE™^23,24^, Withings Healthmate^25^, and CHDR Promasys ePro smartphone applications were installed on the participant’s Android smartphones. The participants were also provided with a Withings Steel HR smartwatch. Training sessions were provided for the Withings devices and the Promasys ePRO application. All participants were monitored for 21 days continuously.

The CHDR MORE™ app enables the unobtrusive collection of data from multiple smartphone sensors (the accelerometer, gyroscope, Global Positioning System, and microphone) and the smartphone usage logs (app usage and calls). The Withings Healthmate app collects data from the Withings devices provided to the participants. The Steel HR smartwatch monitors the participants heart rate, sleep states, and step activity. The ePro app prompted participants to fill in the Positive and Negative Affect Schedule (PANAS) twice daily and depression, anxiety and stress scale-21 (DASS-21) weekly. PANAS is a validated self-reported, brief and easy to administer, 20-item questionnaire that assess positive and negative affect^26^. DASS-21 is a validated self-reported, 21-item measure of three negative emotional states: depression, anxiety and stress^27,28^. More information about the apps and their respective sensors and features can be found in Supplementary Table 1.

### Clinical assessments

The structured interview guide for the hamilton depression scale and inventory of depressive symptomatology (SIGHD-IDSC) assessments were conducted weekly (Day 7, 14, and 21) for all participants in-person at CHDR by trained raters. The SIGHD-IDSC is a single and multi-faceted, and therefore efficient, assessment of depression. The SIGHD-IDSC interview is a combination of the 17-item Hamilton Depression Rating Scale (SIGH-D) and the 30-item Inventory of Depressive Symptomatology-Clinician Rated (IDS-C)^29,30^. The SIGH-D assesses single symptoms on a continuous scale. It is a multidimensional scale that assesses a profile of factors relating to agitation, anxiety (psychic and somatic), guilt, libido, suicide, work, and interest^31^. However, the 17-item scale is still limited in terms of scope. Some symptoms which are often associated with depressed behaviors (such as hypersomnia, weight gain, and reactivity of mood) are not rated^32^. The IDS-C provides additional ratings relating to anxiety, anhedonia, mood, cognitive changes, and vegetative symptoms (relating to sleep, appetite, weight, and psychomotor changes)^32^. Hence, we included the IDS-C as a complementary assessment to provide a broader assessment of depressive symptomatology. IDS-C has been shown to have a higher sensitivity to detect changes in depression severity, therefore deeming it more advantageous for monitoring changes in symptom severity, especially for depression-related drug trials^33^.

### SIGHD-IDSC dimensions

For this study, we investigated the correlation between the remotely monitored features with the total depression severity scores (SIGHD-IDSC) and the scores of individual symptom dimensions. Multiple approaches can be taken to transform the raw data, collected from smartphones and wearable devices, into clinically relevant features. As illustrated by Mohr et al. raw sensor data can be converted in low-level features and high-level behavioral markers^34^. These features and behavioral markers can be used to identify a clinical state or disorder. Low-level features represent descriptive activities, such as time spent at home and total calls per day. High-level behavioral markers can reflect cognition (e.g. distractibility), behaviors (e.g. social avoidance), and emotions (e.g. depressed mood), which can be measured or estimated by the low-level features. For this study, we developed low-level features (e.g. total number of steps per day) that we correlated directly with the clinical state (i.e. depression severity) and to create high-level behavioral markers (e.g. mood) that could be correlated with the clinical state (as described in Supplementary Table 2).

In Table 1, we defined the high-level behavioral markers as SIGH-IDSC symptom dimensions. The categorizations were manually grouped based on their conceptual similarities. In total, the authors created 15 dimensions relating to Agitation, Anxiety (Psychic), Anxiety (Somatic), Guilt, Hypochondria, Interpersonal relationships, Mood, Retardation, Sex, Sleep, Somatic (General), Somatic (Gastrointestinal), Suicidal Ideation, Weight, and Work. In addition, the authors defined global dimensions as the total scores of SIGH-D, IDS-C, and SIGHD-IDSC (the SIGH-D and IDS-C combined) individually.

**Table 1:** Overview of the SIGHD IDS-C symptom and global dimensions and their associated SIGH-D and IDS-C questions.

SIGHD-IDSC symptom dimensions	SIGH-D	IDS-C
Agitation	09. Agitation	24. Psychomotor agitation
Anxiety (psychic)	10. Anxiety (psychological)	06. Mood (Irritable)
		07. Mood (Anxious)
		27. Panic/phobic symptoms
Anxiety (somatic)	31. Anxiety (somatic)	26. Sympathetic arousal
Guilt	02. Feelings of guilt	
Hypochrondia	15. Hypochondriasis	
Insight	17. Insight	
Interpersonal relationships		29. Interpersonal sensitivity
Mood	01. Depressed mood (sad, hopeless, helpless, worthless)	05. Mood (sad)
		08. Reactivity of mood
		09. Mood variation
		10. Quality of mood
		16. Outlook (self)
		17. Outlook (future)
Psychomotor retardation	08. Retardation; psychomotor	23. Psychomotor slowing
Sexual function	14. Genital symptoms	22. Sexual interest
Sleep	04. Insomnia (early)	01. Sleep onset insomnia
	05. Insomnia (middle)	02. Mid-nocturnal insomnia
	06. Insomnia (late)	03. Early morning insomnia
		04. Hypersomnia
Somatic (general)	12. Somatic symptoms general	20. Energy/Fatigability
		25. Somatic complaints
		30. Leaden paralysis / physical energy
Somatic (gastrointestinal)	12. Somatic symptoms (gastrointestinal)	11. Appetite decreased
		12. Appetite increased
		28. Gastrointestinal
Suicidal ideation	03. Suicide	18. Suicidal Ideation
Weight	16. Loss of weight	13. Weight decreased
		14. Weight increased
Activity/reward/hedonic tone	07. Work and activities	15. Concentration/decision making
		19. Involvement
		21. Pleasure/enjoyment
Global dimensions	SIGH-D global score: sum of all SIGH-D dimension scores	IDS-C: Sum of all IDS-C dimension scores

SIGH-D IDS-C: sum of SIGH-D and IDS-C.

### Data pre-processing

All data were inspected and preprocessed using Python (version 3.6.0) and the Pyspark (version 3.0.1) library. Raw data were inspected for missing data, outliers, and normality by the authors AZ and RJD. Missing data were defined as the absence of data for periodic features on a given day or given week (e.g. weight, blood pressure, and the DASS). No missing data definition was provided for the aperiodic activities (e.g. phone calls) as there was no method to distinguish between missing data or no activity. As we used weekly aggregates for the modelling (see the “Feature engineering” section for more information), missing values were not imputed. The advantage is that when missing data are limited to a small number of observations, we can still achieve a comprehensive analysis with incomplete data without adjustment. The disadvantage is that if participants were missing several days of data within one week, then the weekly aggregate would be biased towards days containing data. Outliers were removed if they were deemed illogical and impossible (such as walking more than 70,000 steps per day). Log- or square root-transformation was applied if the distribution of the feature was not normally distributed.

### Feature engineering

The features were provided by the Withings devices and CHDR MORE app at different sampling frequencies (varying from each interaction to every 10 min). Feature engineering is the process of selecting and transforming features from raw data to extract and identify the most informative set of features. These engineered features represent a summarized measure of the collected data. For this study, cumulative parameters, such as step count, were summated per day per subject. Averaged features, such as the heart rate (average beats per minute), which was provided every 10 min, were averaged per day per subject. Supplementary Table 1 illustrates how all the features were aggregated for each data type. The design of these features was based on available data provided by the smartphone and wearable devices, and on a previous published study that had a similar protocol^35^.

Initially, we considered the possibility of integrating multiple data types to create interaction or composite features, such as combining heart rate and steps to determine the heart rate response to activity. However, introducing such interaction features would substantially increase the feature space, making the model more complex and potentially harder to interpret. Given these considerations and the inherent challenges of managing a large feature space, we decided to only rely on the individual features. This approach allowed us to maintain a balance between the dimensionality of the information and the manageability of the model’s complexity.

SIGHD-IDSC scores represent the depression severity over the last week. To create a dataset that is representative of activity over the last week, we transformed the daily activities into weekly averages. Hence, each patient and control had three data points, each point representing an average day in a single week. We have defined a “week” as 6 days prior to the SIGHD-IDSC assessment and the day of the SIGHD-IDSC assessment.

### Feature selection

Feature selection is the process of identifying relevant features that can be used for the model construction. The elimination of irrelevant features would increase the interpretability of the final statistical models^36^. Typically, domain knowledge plays a pivotal role in selecting the most relevant features. However, domain knowledge may not be sufficient when dealing with a multi-dimensional dataset. Hence, automatic feature selection techniques can be used to remove features that are highly correlated, exhibit low variance, or provide a limited amount of information about the dependent variable^37,38^. Prior to the feature selection, 61 features were provided by the CHDR MORE™ and ePro platform (as seen in Supplementary Table 2). The number of features was reduced in a two-step approach. First, we used domain knowledge to eliminate features. We visually inspected features to remove features which exhibited a high degree of missing data (e.g. if the majority of subjects had missing values or had no data) or had limited clinical relevance (e.g. time spent on the ‘comics’ apps category was deemed irrelevant). Second, we used and compared three automated feature selection techniques: Correlation-based feature selection^39^, variance thresholding^40^, and variance thresholding in combination with variance inflation factor (VIF)^41^. Each feature selection technique was used to select a subset of relevant features (based on the weekly aggregated features) and these features were subsequently fitted to the regression models (see Sect. “Statistical analysis”).

### Statistical analysis

#### Estimation of SIGHD-IDSC

R (version 3.6.2) was used for the statistical analysis. While the Pearson’s correlations are typically employed to estimate the correlation coefficient between two outcome variables, correlation coefficients in longitudinal settings (with possible missing values) cannot be obtained with this approach. Hence, we used Linear Mixed-Effects models (LMM) to account for the between- and within-subject variation over time.

We compared the LMM from the lme4 R package^42,43^ and the generalized linear mixed models with L1-penalization from the glmmLasso R package^44^. The glmmLasso models allow for further feature selection by reducing the weight of irrelevant features to zero^45^. As seen in Eq. (1), each of the employed LMMs included a subject-specific random effect to account for the intra-subject correlations between the dependent and independent variables. All other variables were included as fixed effects. No interaction terms were included in the model as we already had more unique features than unique participants, adding more interaction terms would only increase the complexity of the model, as observations within participants may be autocorrelated. To assess if model assumptions were met, each model was visually inspected using quantile–quantile (Q-Q) plots^46^.

Equation (1) Depression severity linear mixed effects model. **Y** is the vector that represents the weekly depression scores. **X** is the fixed effects design matrix, which includes columns for the intercept and the features. **Z** is the random effects design matrix, which includes columns for the subject-specific random effects. ${\varvec{\beta}}$ and **b** represent the vectors for the fixed effects and subject-specific random effects coefficients respectively. ${\varvec{\varepsilon}}$ represents the vector of the independent and identically distributed (I.I.D.) error terms.

1 $$Y=X\beta +Zb+ \varepsilon ,$$

While a LMM of the SIGHD-IDSC total score would provide a broad assessment of depression severity, LMMs of the SIGHD-IDSC dimension scores would provide insights into an individual’s depression symptom profile. In total, we developed 18 LMMs, one for each of the global dimension scores, SIGH-IDSC total score, SIGH-D total score, IDS-C total score, and one for each of the SIGH-IDSC symptom dimensions scores (as seen in Table 1). We did not develop a LMM for the Insight dimension as there was no variation in this assessment during the study period and only one participant had a score of one (the remaining participants had a score of zero).

All LMMs were validated using a repeated nested stratified shuffle split 100 outer-fold (and 50 inner-fold) cross-validation. Cross-validation is a resampling method to assess the generalizability of a statistical model^47^. Nested cross-validation consists of having two non-overlapping cross-validation layers. The inner cross-validation loop optimizes the model configuration, and the outer cross-validation loop assesses the performance of the model generated in the inner loop^48^. In each outer loop, 80% of the data was used for model training, while the remaining 20% was used for model validation. For each loop, all features were standardized (by scaling to the unit variance after subtracting the mean), using the training data only. The 80% training data in the outer loop was used for the train and test split in the inner loop. By using stratification, each dataset split had the same distribution of patients and controls in each fold. This approach mitigates the risk of biased model evaluation due to class imbalance. The limitation of nested cross-validation is that the validation procedure generates a model for each outer-fold. For this study, we reported the average R^2^ and RMSE (root mean square error) of the 100 outer-fold models. The R^2^ represents the percentage of variance that is explained by the remotely monitored features. The RMSE represents the standard deviation of the error between the true depression severity scores from the predicted depression severity scores.

For each SIGH-D IDSC dimension, we applied two types of models. One model contained both the self-reported outcomes and the passively collected features and the other model contained only the passively collected features, hence no DASS or PANAS. By comparing the results from the two models, researchers can assess whether the passively collected data significantly contributes to the predictive power or additional insights into the depression symptoms beyond what can be gathered from the DASS and PANAS alone. In addition, we compared the LMMs to a null model using ANOVA to determine whether the remotely monitored features were significant in predicting the SIGH-D IDSC dimensions. This evaluation aimed to ascertain the significance of the remotely monitored features in predicting the SIGH-D IDSC dimensions. A significant difference in this comparison would indicate the substantial contribution of the remotely monitored features in estimating the severity of depression.

#### Training LMMs with 1,2, and 3 weeks of data

For the secondary objective, we investigated how the quantity of data points used for training influences the model’s performance. In other words, we want to see if using more or fewer data points can improve or hinder the model's accuracy. To do so, we trained the regression models on the first week, the first two weeks, and three weeks of data. Here, we assume that an individual’s week-to-week behavior is habitual and therefore one week of data would constitute a minimally sufficient dataset for model building. We adopted a weekly aggregation approach for each model, where the data were aggregated on a weekly basis. Specifically, for the week 1 model, we had one aggregated weekly observation per subject. As for the week 2 models, we expanded the observations to two aggregated weekly data points per subject. For the training of the LMMs, the dependent variable was the SIGHD-IDSC scores for each week. For the evaluation of the model for the hold-out dataset, the dependent variable was the SIGHD-IDSC for the third week of data (as shown in the Supplementary Fig. 1). As shown in the Supplementary Fig. 1, we validated the performance of the models using a hold-out validation dataset consisting of the third week of data. To ensure that there was no data leakage between the training and validation datasets, we used 70% of the participants for the training dataset, and the remaining 30% for the validation dataset. The dataset was stratified based on the depression symptom severity to ensure that the population distribution was the same in each training and validation datasets. To assess the generalizability of the regression models, we applied 100 outer-fold (50 inner-fold) nested cross validation, with each of the inner-folds creating the optimal regression models based on the training datasets and outer-folds consisting of the third week validation dataset.

## Results and discussion

### Participant characteristics

**Figure 1:**
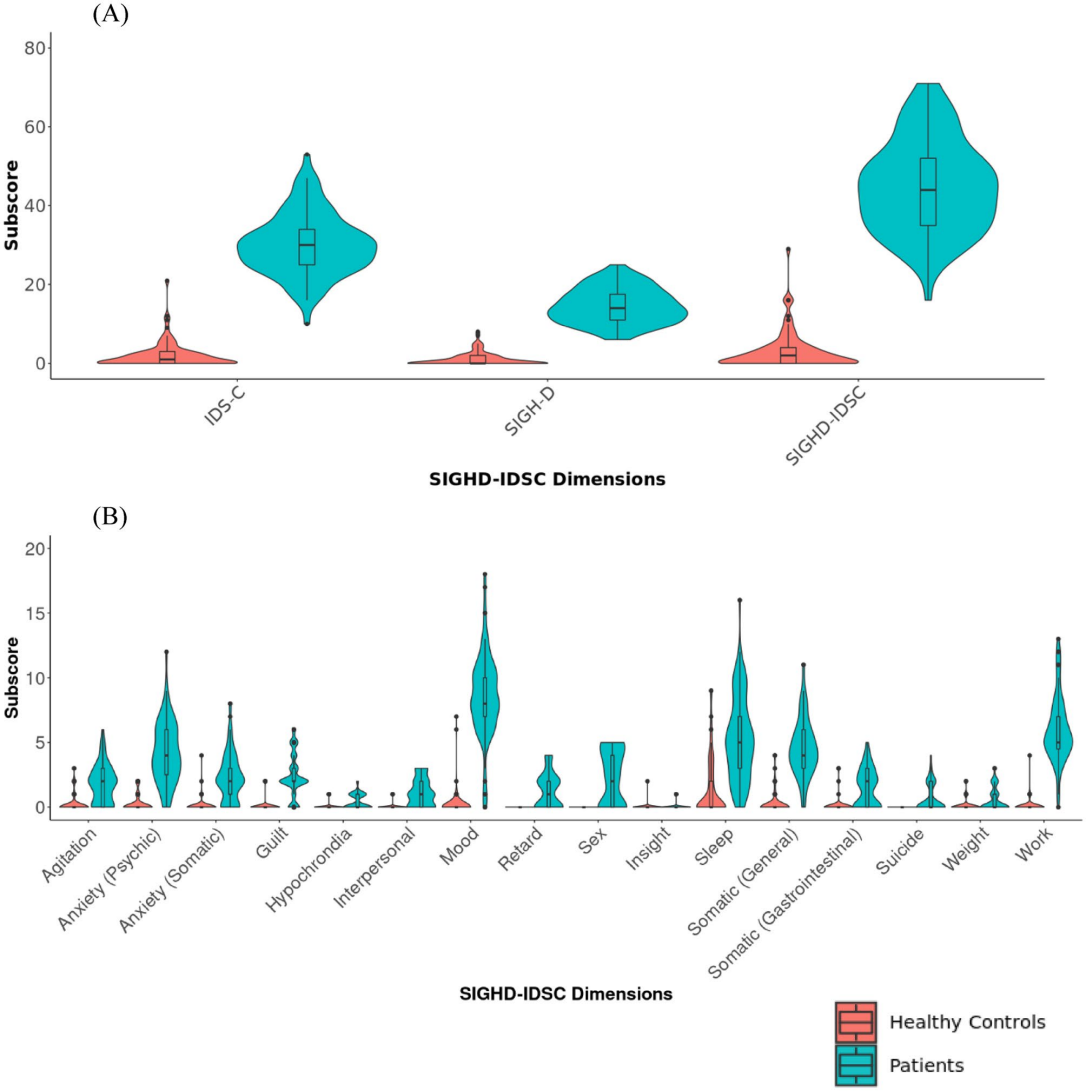
(**A**) Distribution of the SIGH-D, IDS-C, and SIGHD-IDSC global dimensions total scores for patients and healthy controls. (**B**) Distribution of the total scores of the SIGHD-IDSC symptom dimensions for patients and healthy controls. In both figures, red represents the healthy controls while blue represents the patients. The lower and upper box boundaries of the boxplots represent the 25th and 75th percentile range respectively. The line within the boxplot represents the median score. The black scatter plots represent the outliers. The width of the violinplot represents the population distribution of each of the scores.

30 patients and 29 healthy controls were enrolled in the study. Data was collected between March 2019 and March 2020. Supplementary Table 3 provides an overview of the demographic characteristics of the enrolled patients and healthy controls. In total, 177 SIGHD-IDSC total scores were collected (3 weeks for all 30 patients and 29 healthy controls). The last healthy control was not included due to the COVID-19 lockdown^49^. The patients had a mean MADRS total score of 29 (and standard deviation of ± 3.5), and MADRS was not collected for the healthy controls as it was only used to screen the unipolar depressed patients. The patients had a mean SIGH-D total score of 14.5 (± 4.5) and a mean IDS-C total score of 30.5 (± 8.5). The healthy volunteers had a mean SIGH-D total score and IDS-C total score of 1 (± 2) and 1(± 3) respectively. Figure 1 illustrates the distribution of the SIGHD-IDS, SIGH-D, IDS-C, and SIGHD-IDSC symptom dimensions total scores for both the patients and healthy controls. To illustrate if the models were able to account the longitudinal variability of the SIGHD-IDSC dimension scores, Supplementary Table 6 shows the average change in depression scores among the unipolar depressed patients and the healthy controls. Given the 3-week observation period, we speculate that this period is insufficient to observe longitudinal variability.

### Data quality

To assess the quality of our data, we examined the number of days, features, and participants with missing data. In Supplementary Table 4, we found that most of the missing data were from the sleep and location features, however the percentage of missing days were less than 5% of the days and related to 12% of the participants. In the case of the DASS, our expectation was to receive 4 responses per person, totaling 236 responses. However, we received only 196 responses, resulting in an 83% completion rate. Similarly, for the PANAS, we anticipated 42 responses per person, amounting to a total of 2478 responses. However, we obtained 1585 responses, indicating a completion rate of 66%. We found that 64% of the 61 features had no outliers, 29% of the features (concerning 15% of the participants) had one outlier, and the remaining 5% of the features (concerning 5% of the participants) had two outliers.

### Performance of LMMs

Among the different feature selection methods and LMMs used, the variance thresholding in combination with the LMM consistently yielded the highest R^2^ and lowest RMSE across all the dependent features. Hence, we only reported the results of these variance thresholding LMM depression severity models. When including both the healthy controls and the patients, and when using both the passively collected features and the self-reported outcomes (the DASS and the PANAS), the SIGH-D, IDS-C, and SIGHD-IDSC LMMs achieved an R^2^ of 0.80, 0.80, and 0.73 and a scaled RMSE of 5.3, 9.9, and 15.1 respectively. Table 2 provides an overview of the performance of the 18 SIGHD-IDSC dimension LMMs. The LMMs with the highest R^2^ were the SIGHD-IDSC dimensions related to mood (0.72) and work (0.65). While the LMMs with the lowest R^2^ were the SIGHD-IDSC dimensions related to retardation (0.40) and hypochondria (0.40). Supplementary Table 1 highlights the advantages of including healthy controls in the LMMs. When examining the predictive performances separately for patients and healthy controls, it is observed that the R^2^ and RMSE are lower compared to when they are combined. However, it is important to note that the overall predictive performance may still be valuable in both cases.

**Table 2:** Performance of the LMM to estimate the total scores of the SIGH-D, IDS-C, SIGHD-IDSC global dimensions, and each of the SIGHD-IDSC symptom dimensions.

SIGHD-IDSC global and symptom dimensions	Marginal R^2^ mean (including DASS and PANAS)	Mean RMSE (including DASS and PANAS)	Marginal R^2^ mean (without DASS and PANAS)	Mean RMSE (without DASS and PANAS)
SIGH-D	0.73 (± 0.01)*	5.30 (± 0.17)	0.20 (± 0.01)*	10.28 (± 0.19)
IDS-C	0.80 (± 0.01)*	9.90 (± 0.32)	0.25 (± 0.01)*	18.58 (± 0.29)
SIGHD-IDSC	0.80 (± 0.01)*	15.1 (± 0.48)	0.24 (± 0.01)	28.33 (± 0.45)
Agitation	0.47 (± 0.01)*	0.99 (± 0.04)	0.22 (± 0.01)	1.83 (± 0.03)
Anxiety (psychic)	0.63 (± 0.01)*	1.70 (± 0.06)	0.21 (± 0.01)*	3.41 (± 0.07)
Anxiety (somatic)	0.57 (± 0.01)*	1.16 (± 0.06)	0.24 (± 0.01)	2.11 (± 0.05)
Guilt	0.57 (± 0.02)*	1.01 (± 0.04)	0.24 (± 0.01)	1.75 (± 0.04)
Hypochrondia	0.40 (± 0.02)	0.27 (± 0.02)	0.30 (± 0.02)	0.47 (± 0.01)
Interpersonal	0.60 (± 0.01)	0.56 (± 0.02)	0.15 (± 0.01)	1.02 (± 0.02)
Mood	0.72 (± 0.01)*	3.04 (± 0.10)	0.23 (± 0.01)*	5.97 (± 0.11)
Retardation	0.40 (± 0.02)*	0.61 (± 0.03)	0.27 (± 0.02)	1.08 (± 0.03)
Sex	0.45 (± 0.02)*	1.01 (± 0.05)	0.20 (± 0.01)	1.94 (± 0.04)
Sleep	0.47 (± 0.02)*	2.34 (± 0.07)	0.29 (± 0.02)	4.43 (± 0.09)
Somatic (general)	0.62 (± 0.02)*	1.88 (± 0.07)	0.31 (± 0.02)	3.51 (± 0.08)
Somatic (gastrointestinal)	0.43 (± 0.02)*	0.71 (± 0.03)	0.18 (± 0.01)	1.50 (± 0.04)
Suicide	0.50 (± 0.01)*	0.32 (± 0.02)	0.28 (± 0.02)	0.57 (± 0.02)
Weight	0.43 (± 0.01)*	0.37 (± 0.02)	0.23 (± 0.01)	0.85 (± 0.03)
Work	0.65 (± 0.01)*	2.02 (± 0.07)	0.26 (± 0.01)	3.92 (± 0.07)

The * represents a statistically significant difference (p < 0.05) between the null model and the best performing LMM models.

Nevertheless, when we confined our analysis to solely the passively collected features, effectively excluding the DASS and PANAS, we noticed a substantial decline in the R^2^ and the statistical significance between the full and null models. Table 2 illustrates that when DASS and PANAS are excluded, the marginal R^2^ values for SIGHD, IDS-C, and SIGHD-IDSC decrease by approximately 55 percentage points. Moreover, the RMSE sees an approximate twofold increase without DASS and PANAS. Additionally, there’s no longer a statistical difference between the SIGH-D IDSC full and null models. This notable decrease underscores the pivotal role that the DASS and PANAS play in predicting the final outcomes of the models. In other words, these self-reported features significantly contribute to the accuracy of our predictive models.

**Figure 2:**
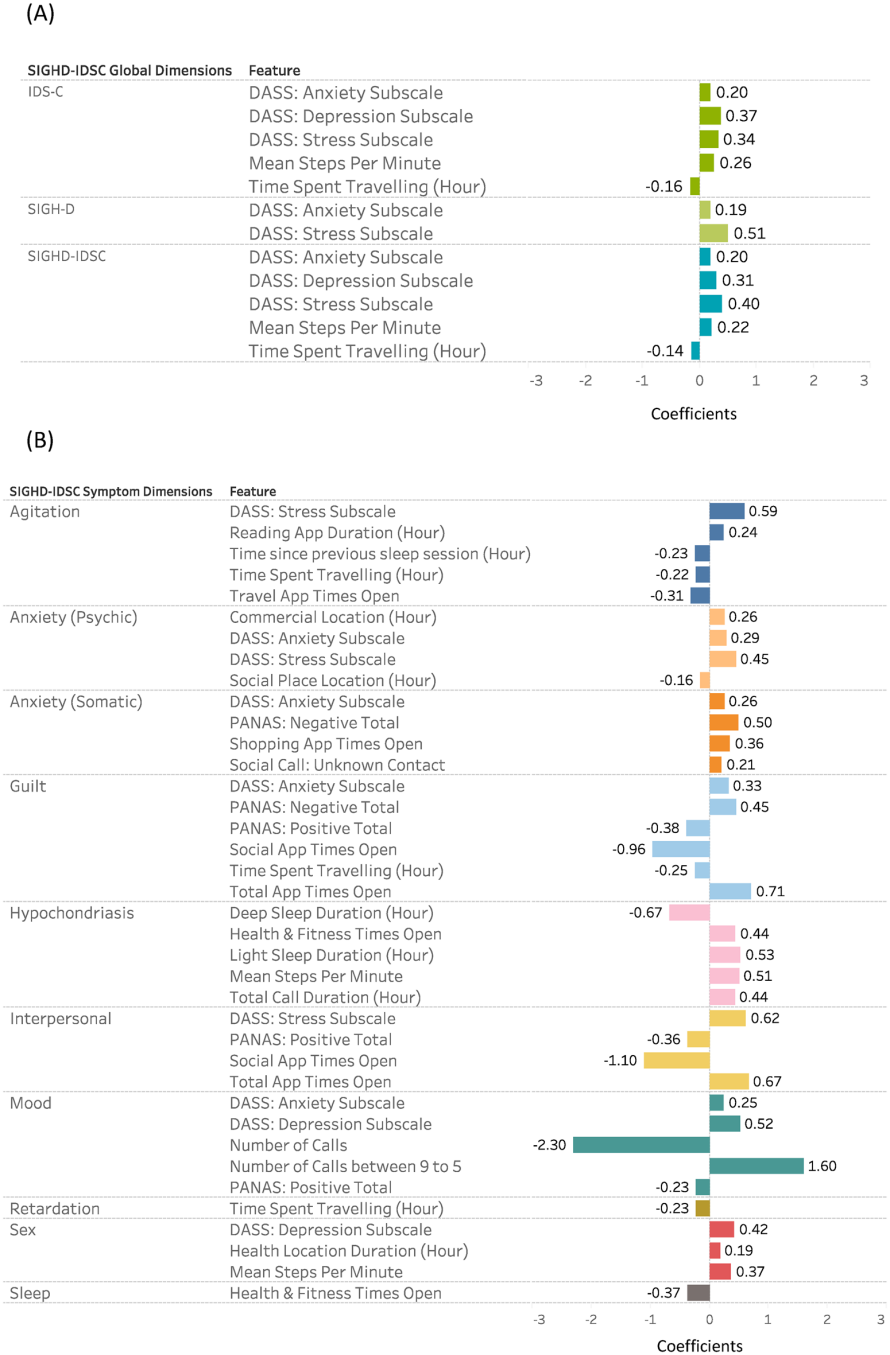
Overview of all significantly correlated features (p < 0.05) for each of the (**A**) SIGHD-IDSC global and (**B**) symptom dimensions. The bars represent the correlation coefficients for each of the significant features. The color of the bars represents each of the SIGHD-IDSC global and symptom dimensions.

However, it's important to also acknowledge the contribution of the passively collected features in this context. While their predictive power might not be as substantial as that of the DASS and PANAS, they still hold relevance. The barplots in Fig. 2 show not only the significant role of passively collected features in predicting outcomes but also show a relatively diminished influence of the PANAS, with SIGH-D being an exception. The figure underscores the combined predictive strength of the full model, encompassing DASS, PANAS, and the passively collected features, isn't solely attributed to the self-reported outcomes, which highlights the value of integrating diverse data sources. These passively collected features likely capture various aspects of the subjects' behaviors and responses that might not be directly accounted for in self-reported data. Their inclusion enriches the overall predictive capability of the models, albeit to a lesser extent compared to the DASS and PANAS. The integration of DASS, PANAS, and passive data ensures a comprehensive reflection of an individual's depressive state, by also evaluating their daily behavioral and physical states. Further the inclusion of self-reported and passive sources of data allows for cross-verification. If both the self-reported data and passive data indicate a similar trend, it strengthens the validity of the findings. Conversely, if discrepancies were to arise, this could prompt further investigations into novel research areas that might need more focused attention.

When we considered both passive data and self-reported features (including DASS and PANAS), our analysis revealed that the majority of the models (with the exception of Hypochondria and Interpersonal dimensions) exhibited a significant divergence from the null models. This divergence implies that the presence of predictors in the full models yielded outcomes that were noticeably different from those of the null models (Table 2). When we solely utilized the passive features, excluding the DASS and PANAS, we only observed that the SIGH-D, IDS-C, Anxiety (Psychic), and Mood models significantly differed from the null models.

The drop in the marginal R^2^ and reduced significant difference between the models that do and do not include the self-reported outcomes underscores the significance of DASS and PANAS in estimating the SIGH-D IDSC dimensions. In other words, the presence or absence of these self-reported features significantly impacts the accuracy of our models in estimating the different dimensions of SIGH. This insight further accentuates the importance of considering self-reported data like DASS and PANAS alongside passive data for more accurate estimations of these dimensions.

### Correlations

For each of the LMMs, we identified the correlation coefficients and their significance between the remotely monitored features and the depression severity scores. As seen in Fig. 2, there was a significantly positive correlation between the mean SIGH-D total score with the DASS-Anxiety and DASS-Stress (p < 0.05). Both the IDS-C and the SIGHD-IDSC total scores were significantly positively correlated with the DASS-Depression, Anxiety, and Stress total scores and significantly negatively (p < 0.05) correlated with the mean steps-per-minute and time spent travelling. We found that the Depression, Anxiety, and Stress total scores (from the DASS) and location features were significantly correlated with 7 (Agitation, Anxiety (Psychic), Anxiety (Somatic), Guilt, Interpersonal, Mood and Sex) and 6 (Agitation, Anxiety (Psychic), Guilt, Hypochondriasis, Retardation, and Sex) of the mean SIGHD-IDSC symptom dimensions respectively.

### Training LMMs with 1,2, and 3 weeks of data

**Figure 3:**
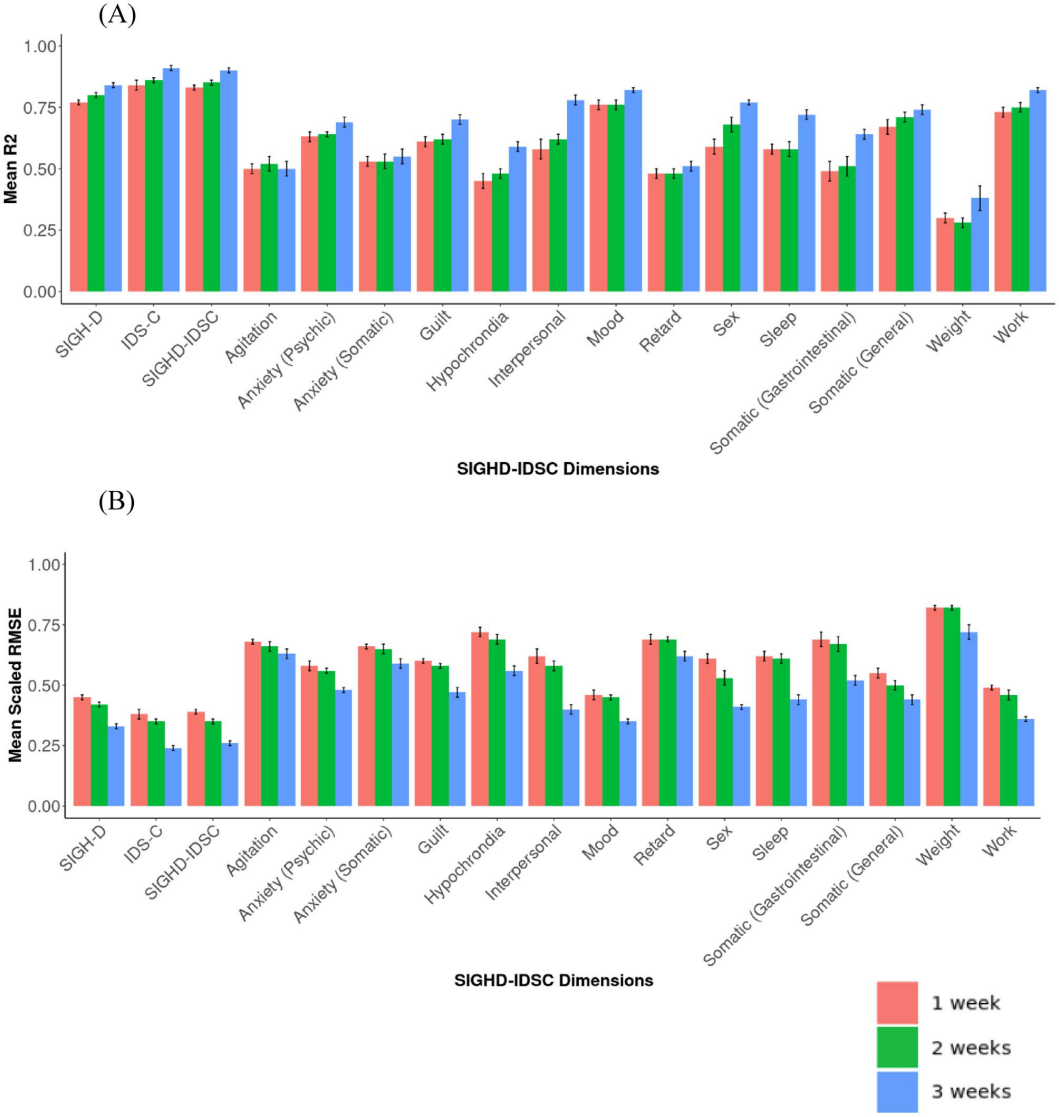
(**A**) and (**B**) represent the mean R^2^ and mean scaled RMSE for each of the SIGHD-IDSC global and symptom dimension LMMs. Each color represents the dataset used for training the models. The error bars represent the standard deviation across each of the 100 outer-fold predictions.

Overall, we found that training the models on three weeks of data consistently yielded the highest R^2^ and the lowest RMSE for each of the SIGHD-IDSC global and symptom dimensions compared to the models trained on the first week and first two weeks of data with the exception one dimension, Agitation (as seen in Fig. 3). For the Agitation dimension, the models trained on the first two weeks of data yielded the highest R^2^. The difference in R^2^ between the first week and the third weeks models was relatively marginal (a difference of 0.07) for the SIGHD-IDSC global dimension. However, the difference in the scaled RMSE between the two models was notable, with a difference of 0.13.

## Discussion

In this pilot study, we provided a comprehensive assessment of the relationship between depression severity and subjective and objective features sourced from data collected by smartphone and wearable devices under free-living conditions. Our results illustrate that, features related to self-reported depression, anxiety scores, stress scores, physical activity, and not social activities, were significantly correlated with depression severity. These features can collectively serve as a composite biomarker to estimate the gold standard in-clinic assessment, the SIGHD-IDSC.

### Data quality

The missing and outlier data only impacted a minority of the participant’s data and did not lead to the exclusion of any weekly aggregated features used in the analysis (Supplementary Table 4). Given the low number of missing data and outliers, we did not observe any differences in data quality between the depressed patients and controls. While we could not identify any similar trials to compare data quality, we deem that our protocol led to the collection of a robust and reliable dataset. However, the aggregation of the data undermines the opportunity to identify potentially nuanced daily behaviors and higher order interactions between multiple features. For example, social and physical activity behavior most likely differs per location and between weekdays and weekends, but these daily interaction features are not reflected in the current dataset. The identification of higher order behavioral patterns or routines per location and per day could enrich the sensitivity of the composite biomarkers.

### Estimation of the SIGHD-IDSC

Our findings indicate that a combination of remotely monitored self-reported and objective features can serve as a composite biomarker to estimate weekly depression severity. The interplay between these two types of features—self-reported and passively collected—is essential for a comprehensive and accurate prediction of the outcomes. The DASS and PANAS, owing to their direct reflection of subjects' mental states, emerge as potent predictors. Concurrently, the passively collected features contribute a nuanced layer of information, further enhancing the models' overall predictive capacity. This underscores the value of a holistic approach that encompasses both types of data sources in generating reliable predictions for the studied dimensions.

We found our approach was better suited for evaluating the global dimensions (SIGH-D, IDS-C, and SIGH-IDSC total scores), rather than the manually defined SIGHD-IDSC symptom dimensions, such as mood, weight, or sex (Table 2). The symptom dimension models were a moderate to strong representation of work, somatic (general), interpersonal, anxiety (psychic) and mood dimensions and a poor representation of the hypochondria and retardation dimensions. This illustrates that the features obtained correspond to some but not all of the SIGHD-IDSC dimensions. One explanation for the limited agreement between the remotely monitored biomarkers and the SIGHD-IDSC dimensions is the comparison of objective measures with subjective assessments. For example, we compared objective sleep measurements (such as sleep duration, and the number of light and deep sleep periods) to the subjective interpretations of sleep quality by the patient or the clinician as reflected in the SIGHD-IDSC. Despite having several objective measures relating to sleep, we found that the sleep model captured less than half of the variance. Previous studies have illustrated that objective sleep assessments are not strongly correlated with subjective reports of sleep^50,51^. Discrepancies between the objective and subjective measures of sleep could be influenced by several factors, such as mood at the time of awakening^52^, insomnia, negative bias, and impaired memory^53^. These findings highlight that those subjective experiences are not always represented by objective measures. Hence, in the context of clinical trials for depression, the identified relevant features are better suited for monitoring overall depression severity rather than monitoring specific depression symptoms.

### Inclusion of healthy controls

The inclusion of health controls in the models provides several benefits. Firstly, by incorporating more participants, the number of observations available for analysis increases. This larger sample size enhances the statistical power of the LMMs, which leads to more reliable and robust predictions. Additionally, the inclusion of healthy controls introduces a broader range of depression severity scores, spanning from zero to minimal symptoms. In addition to enhancing the model's ability to capture the full spectrum of depression severity and improving its generalizability, the wider range of scores also allows for the inclusion of potential remission in depressed patients. As their scores move towards zero, the model can accurately capture the possibility of their condition improving and reaching a state of remission.

### Correlation with the SIGHD-IDSC dimensions

Both the self-reported DASS and daily travel routines were consistently significantly correlated with the SIGH-D, IDS-C and SIGHD-IDSC global dimension total scores (Fig. 2). More specifically, we found that depression, anxiety, and stress total scores were positively correlated with overall depression severity. Additionally, participants with higher depression scores tended to walk at a faster pace but spent less overall time in transit. This means that while they moved more quickly, their total travel duration was shorter. Our findings are supported by previous studies that found correlations between both smartphone-based self-reported assessments and location-based behaviors^16,54,55^ with in-clinic depression rating scales^13,56,57^. Notwithstanding, we have not identified any research that supports the notion that unipolar depressed patients have increased walking speeds, rather, the current literature suggests that depressed patients exhibit more motor disturbances and thus reduced walking speeds^58^. However, these inferences were based on instrumented gait assessments performed in controlled settings, and not based on real-world evidence. This implies that inferences regarding gait or other motor disturbances assessed in the clinic may not always correspond with behaviors outside the clinic. Together, our findings highlight the importance of collecting both self-reported subjective and objective behavioral features, such as DASS, gait and travel patterns, in depression drug trials as they represent a more holistic biomarker of depression. Further, behaviors characteristic to depression that were identified within a clinical setting may not correspond to behaviors exhibited outside a clinical setting.

### Number of weeks of data for training

Our findings indicate that the models overall performed better when trained on three weeks of data, rather than one or two weeks (Fig. 3). However, for the SIGHD-IDSC global dimensions, the difference in the variance explained between the first week and three weeks of data was marginal. While the inclusion of three weeks of data notably reduced the prediction error. Depending on the mechanism of action of any given antidepressant drug, therapeutic effects may only become evident after several weeks of treatment with, for example SSRIs, or may rapidly occur and then dissipate over a week or two as with the NMDAR antagonist ketamine^59,60^. It is therefore crucial to determine how long and how often patients need to be monitored to extract reliable and meaningful inferences from the data following an intervention. Collecting excessive data can be time-consuming and resource-demanding, however having insufficient data can undermine the accuracy of the extrapolations. Although the present study was of non-interventional nature, this suggests that a minimum of three weeks of data are required to create a representative dataset that would build an accurate model that represents depression severity in future interventional trials. However, the trade-off between number of weeks used for training and the model performance was marginal.

### Limitations

There are several limitations to our approach. Due to the small sample size, relatively short observation period, and the number of technical devices used (Android smartphone and Withings wearables), there is a limited understanding of what degree our findings are generalizable to other cohorts, technical devices, and clinical assessments. A follow-up study is needed to assess how well our findings can translate to other depressed patients whose data are collected in a different time period using different devices (such as an iPhone and Apple Watch). Further, given the limited agreement between the objective measures of sleep and the SIGHD-IDSC sleep dimension scores, a follow-up study may choose to incorporate both objective and subjective measures of sleep such as polysomnography and self-report questionnaires related to sleep to further improve the reliability of the features.

### Application

Previous studies of this nature have primarily focused on predicting self-reported outcomes; however, our study emphasized the value of predicting the clinical assessments of depression. By combining both self-reported assessments and passively collected data, our models seek to predict a psychiatrist's weekly assessment of depression. We believe this approach holds greater promise for real-world utility since it considers not just the individual’s perception, but also a clinician's assessment, which is typically based on a broader spectrum of indicators and professional expertise. Furthermore, predicting a psychiatrist's assessment using a combination of self-reports and passive sensing data bridges the gap between subjective patient insights and objective clinical evaluations. By doing so, we provided a more comprehensive tool that might be more easily integrated into clinical workflows, thereby advancing beyond feasibility to actual deployment.

Based on our findings, remotely monitored features cannot substitute the clinical assessment of depression severity. However, our approach can potentially serve as a complementary tool to assess clinical symptoms of depression over time in free-living conditions. This approach towards collecting granular data can offer insights into subtle changes that might not be immediately apparent through traditional assessment methods. For instance, if a new antidepressant is being tested, rather than relying solely on periodic self-reports from patients about their mood or well-being, the tool can track behavioral metrics (like activity levels or sleep patterns) that might shift with changes in mood or medication efficacy. Further, a common pitfall with self-reported outcomes is their reliance on a patient's memory and perception. Patients might forget certain events, underreport symptoms, or be influenced by recent events. By complementing these self-reports with continuous, objective data from our proposed methodology, clinicians and researchers can gain a fuller picture. This blend of subjective self-report and objective behavioral data ensures a more rounded, continuous perspective on a patient's condition, allowing for more informed decision-making in both treatment and research settings. Remotely monitored composite biomarkers therefore are strong candidates for filling-in and complementing the retrospective gaps that are typical of in-person clinical assessments. Hence our approach is expected to benefit drug development for mood disorders, since it could aid the monitoring and assessment of depression severity during clinical trials based on both in-clinic rater-based interviews and out-of-clinic activities and self-reported outcomes.

## Conclusion

We presented a novel approach to monitoring depression severity among unipolar depressed patients using data sourced from smartphone and wearable devices. In this longitudinal non-interventional study, we collected a relatively robust dataset, consisting of few missing data points and outliers. We identified the relevant smartphone- and wearables-based features that collectively create a biomarker that could estimate the SIGH-D, IDS-C and SIGHD-IDSC global and symptom dimension total scores. Together, these findings suggests that objective and subjective features captured by these remote monitoring devices can collectively serve as a composite biomarker to estimate depression severity under free-living conditions.

### Supplementary Information

Supplementary Figure 1.

Supplementary Figure 2.

Supplementary Table 1.

Supplementary Table 2.

Supplementary Table 3.

Supplementary Table 4.

Supplementary Table 5.

Supplementary Table 6.

## Data Availability

Data can be made available upon reasonable request.
