# Supplementary material for: A smartphone- and wearable-based biomarker for the estimation of unipolar depression severity
Source: Sci Rep. 2023 Nov 1;13:18844. doi: 10.1038/s41598-023-46075-2 (PMC10620211; doi:10.1038/s41598-023-46075-2)
Supplement: Supplementary file 1 — Supplementary Figure 1. [file 41598_2023_46075_MOESM1_ESM.docx]

This step is repeated across 100 iterations with a different combination of subjects in each split

Supplementary Figure 1 Diagram for the 100-fold nested cross validation for the estimation of the third week of SIGH-IDSC global and symptom dimension total scores.
