# Supplementary figures and images for: A smartphone- and wearable-based biomarker for the estimation of unipolar depression severity
Source: Sci Rep. 2023 Nov 1;13:18844. doi: 10.1038/s41598-023-46075-2 (PMC10620211; doi:10.1038/s41598-023-46075-2)

| **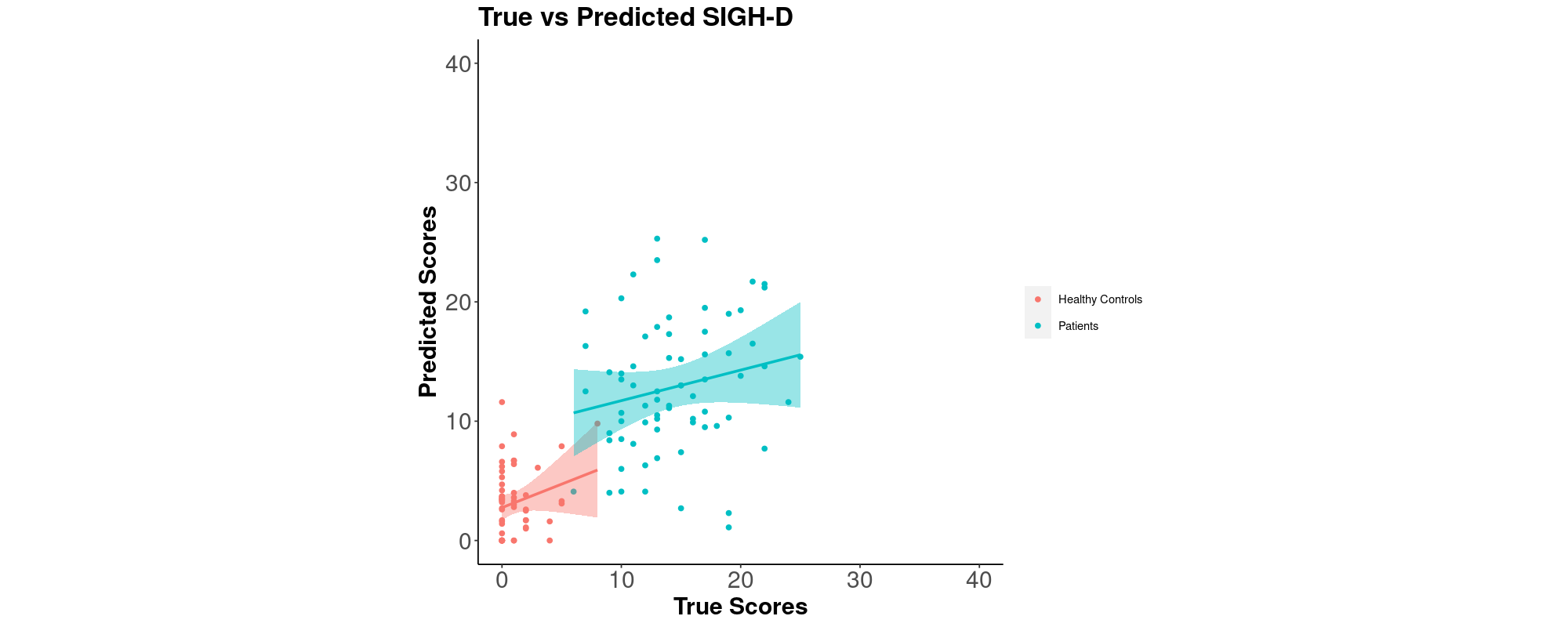** | 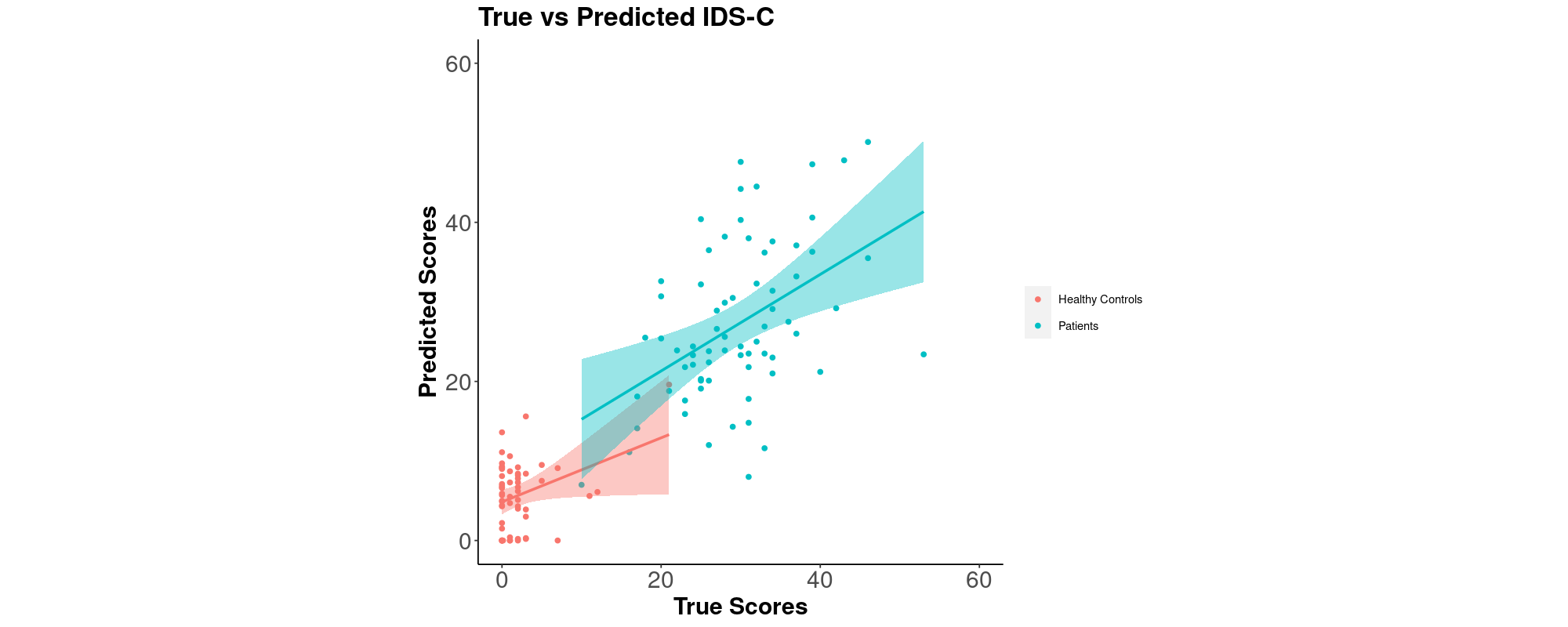 |
| --- | --- |
| 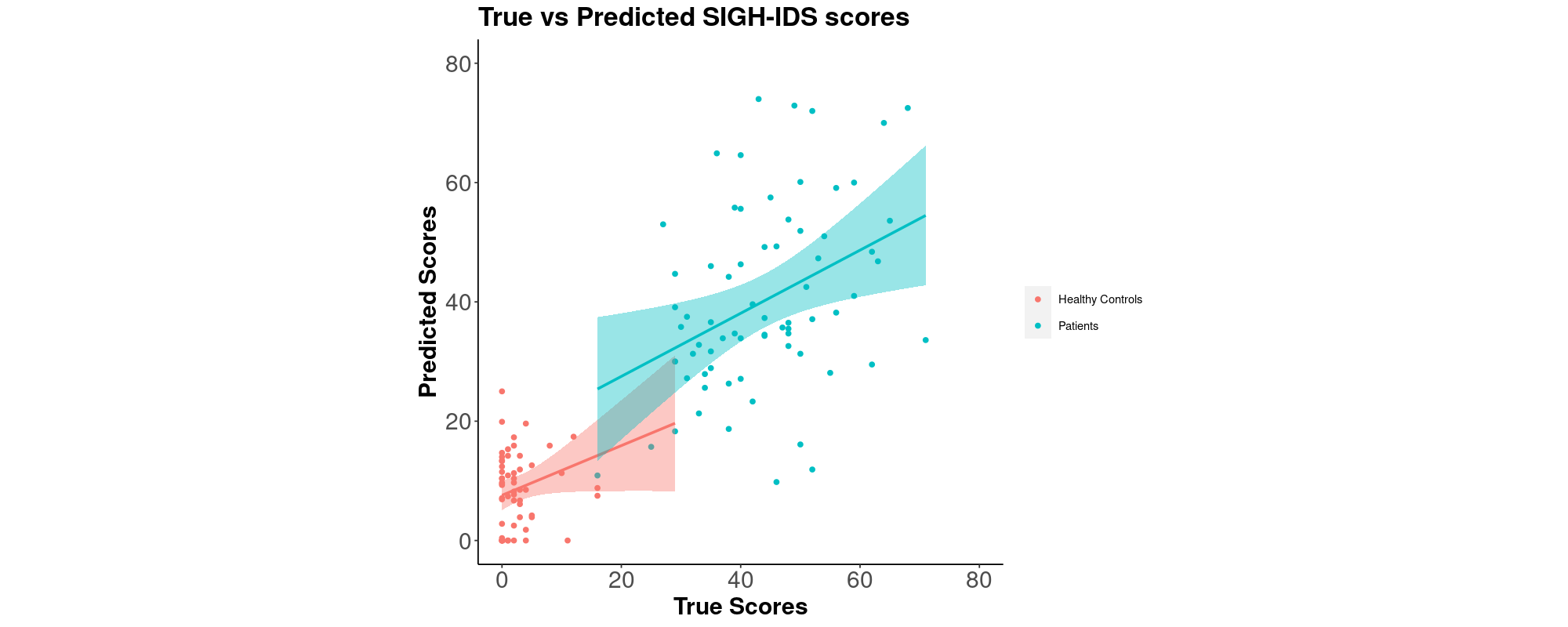 | 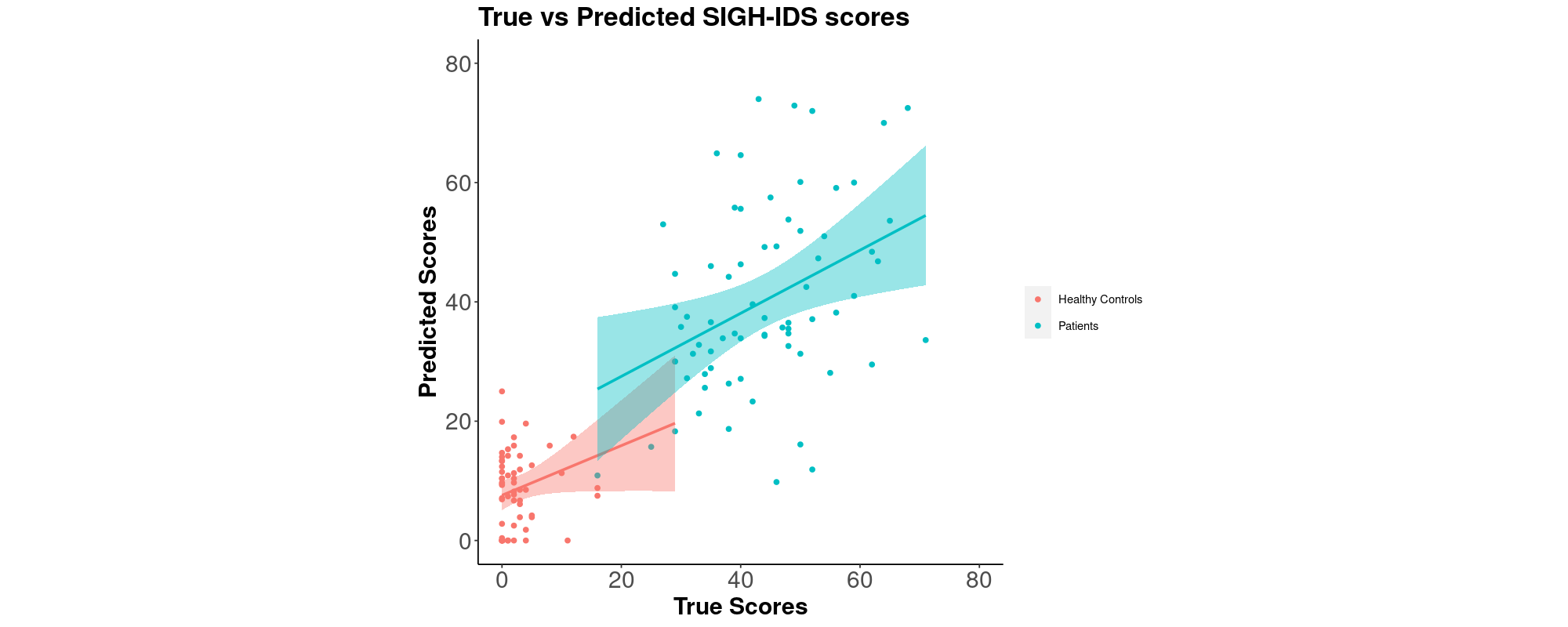 |

Supplementary Figure 2 Comparison of the true and predicted SIGH-D, IDS-C, and SIGH-D IDS-C scores.

Supplement: Supplementary file 2 — Supplementary Figure 2. [file 41598_2023_46075_MOESM2_ESM.docx]
