# Supplementary material for: A smartphone- and wearable-based biomarker for the estimation of unipolar depression severity
Source: Sci Rep. 2023 Nov 1;13:18844. doi: 10.1038/s41598-023-46075-2 (PMC10620211; doi:10.1038/s41598-023-46075-2)
Supplement: Supplementary file 3 — Supplementary Table 1. [file 41598_2023_46075_MOESM3_ESM.docx]

Supplementary Table 1 A summary of how the features were aggregated based on the data type.

| **Data Type** | **Time Unit** | **Example Feature** | **Aggregation Format** | **Example Aggregation** |
| --- | --- | --- | --- | --- |
| **Count** | Per day | Steps | Sum  Mean  Max | Total Steps  Max Steps Per Hour  Mean Steps Per Hour |
| **Continuous Data within a Range** | Per day | Heart Rate | Min (5%)  Median (50%)  Max (95%) | Lowest 5% Heart Rate  Median Heart Rate  Maximum 95% Heart Rate |
| **Duration** | Per day | App Usage | Total Duration    Mean Duration | Total Duration of Social Apps Opened  Mean Duration of Social App Opened Per Instance |
| **GPS Coordinates** | Per day | Location | Sum  Max  Mean | Total Distance Travelled  Mean And Max Distance From Home |
