# Supplementary material for: A smartphone- and wearable-based biomarker for the estimation of unipolar depression severity
Source: Sci Rep. 2023 Nov 1;13:18844. doi: 10.1038/s41598-023-46075-2 (PMC10620211; doi:10.1038/s41598-023-46075-2)
Supplement: Supplementary file 4 — Supplementary Table 2. [file 41598_2023_46075_MOESM4_ESM.docx]

Supplementary Table 2 An overview of the CHDR MORE^TM^ extracted features.

| **Category** | **MORE Features** | **Derived Features** | **Excluded Features** |
| --- | --- | --- | --- |
| **Demographics** | Age; Gender |  |  |
| **Acceleration**  **(Smartphone)** | Acceleration Magnitude  Gyroscope  Magnometer | 98% Acceleration Magnitude | Mean Acceleration Magnitude |
| **Activity**  **(Smartphone)** | Steps  Heart Rate  Physical Activity Duration  Calories | Steps:  Total Steps, Max Steps Per Hour, Mean Steps Per Hour    Heart Rate:  5%, 50% & 95% Beats Per Minute (BPMs), Standard Deviation of BPMs, % Time Spent In Resting State    Physical Activity:  Soft, Moderate and Intense Activity Duration | Calories  Distance Travelled  Distance Per Step |
| **Apps**  **(Smartphone)** | App Categories:  Communication & Social  Health & Fitness  Recreational  Shopping  Tools  Travel | Duration  Times Open | House & Home App  Libraries & Demo App  Reading App  All duration features |
| **BODY**  **(WIthings)** | Diastolic Blood Pressure  Systolic Blood Pressure  ​Heart Pulse (Bpm)​  Weight​ |  | Height (M)​  Fat mass (kg)​  Fat ratio (%)​  Hydration​  Muscle Mass​ |
| **Location**  **(Smartphone)** | Location Categories:  Commercial  Health  Home  Leisure  Public  Social  Travel | Total Duration at Place​  Total Distance Travelled  Total No of Unique Places Visited  Max Distance from Home  Time Spent Commuting |  |
| **Social**  **(Smartphone)** | Calls  Voice | Number of Calls  Number of Unique Numbers  Number of Incoming, Outgoing and Missing Calls  Number of Calls from Known and Unknown Numbers  Total Duration of Calls  Average Duration of Calls  % Time Human Voice Is Detected | Text messages (SMS) |
| **Sleep**  **(Withings)** |  | Number of Sleep Sessions  Total Sleep Duration  Number of Sleep Phases (Awake, Light Sleep And Deep Sleep)  Duration of Sleep Phases (Awake, Light And Deep Sleep)  Time Between Sleep Sessions  Time to Fall Asleep |  |
| **Epro**  **(Smartphone)** | Self-assessments | Twice daily PANAS  Weekly DASS-21 |  |
