# Supplementary material for: A smartphone- and wearable-based biomarker for the estimation of unipolar depression severity
Source: Sci Rep. 2023 Nov 1;13:18844. doi: 10.1038/s41598-023-46075-2 (PMC10620211; doi:10.1038/s41598-023-46075-2)
Supplement: Supplementary file 5 — Supplementary Table 3. [file 41598_2023_46075_MOESM5_ESM.docx]

Supplementary Table 3 An overview of demographic characteristics of the enrolled patients and healthy controls

| **Demographics** | **Descriptor** | **Patients** | **Healthy controls** |
| --- | --- | --- | --- |
| **Gender** | Female  Male | 24  6 | 25  4 |
| **Race** | African American or Black  Asian  Mixed  Other  White | 2  2  4  1  21 | 1  3  0  1  25 |
| **Age** | Mean (STD)  [Min, Max] | 35(13)  [18, 64] | 35(13)  [20, 63] |
| **BMI (kg/m^2^)** | Mean (STD)  [Min, Max] | 24(3)  [20, 31.5] | 24(3)  [18, 31] |
| **MADRS** | Mean (STD)  [Min, Max] | 29 (4)  [23, 38] | N/A |
| **SIGH-D Total** | Mean (STD)  [Min, Max] | 14.5(4.5)  [6, 25] | 1(2)  [0, 8] |
| **IDS-C Total** | Mean (STD)  [Min, Max] | 30.5(8.5)  [10, 62] | 1(3)  [0,21] |
| **SIGH-IDSC Total** | Mean (STD)  [Min, Max] | 45(12)  [16, 71] | 3(5)  [0,29] |
