# Supplementary material for: A smartphone- and wearable-based biomarker for the estimation of unipolar depression severity
Source: Sci Rep. 2023 Nov 1;13:18844. doi: 10.1038/s41598-023-46075-2 (PMC10620211; doi:10.1038/s41598-023-46075-2)
Supplement: Supplementary file 6 — Supplementary Table 4. [file 41598_2023_46075_MOESM6_ESM.docx]

Supplementary Table 4 A summary table of the number of missing days or days containing excluded outliers and number of participants with missing or outlier days are shown. Features with no missing data or excluded outliers are not shown. For aperiodic features, it is not possible to differentiate between missing data and no data, thus missing data for these features are not represented.

| Feature Category | Feature | no. of days with missing data | NO. of participants with missing data days | No. of excluded outliers | | NO. of participants with excluded outliers |
| --- | --- | --- | --- | --- | --- | --- |
| Accelerometer | Acceleration Magnitude 98% | 1.12 | 2 | | 0 | 0 |
| Apps | Times Open Shopping App | - | - | | 2 | 1 |
|  | Times Open Travel App | - | - | | 1 | 1 |
|  | Total Times App Open | - | - | | 1 | 1 |
| Calls | Calls from known contact | - | - | | 1 | 1 |
|  | Missed Calls | - | - | | 1 | 1 |
|  | Calls from unknown contact | - | - | | 1 | 1 |
| Location | Total unique places visited | 4.49 | 7 | | 1 | 1 |
|  | Total km travelled | 3.93 | 5 | | 1 | 1 |
|  | Time spent travelling | 3.93 | 5 | | 1 | 1 |
| Steps | All steps parameter | 1.69 | 3 | | 1 | 1 |
| Heart Rate | All heart rate parameters | 2.25 | 4 | | 0 | 0 |
| Sleep | Light and deep sleep duration and count | 3.93 | 7 | | 0 | 0 |
|  | Longest sleep session | 3.93 | 7 | | 0 | 0 |
|  | Time since previous sleep session | 3.93 | 7 | | 1 | 1 |
|  | Time to fall asleep | 3.93 | 7 | | 1 | 1 |
|  | Total sleep duration | 3.93 | 7 | | 0 | 0 |
