# Supplementary material for: A smartphone- and wearable-based biomarker for the estimation of unipolar depression severity
Source: Sci Rep. 2023 Nov 1;13:18844. doi: 10.1038/s41598-023-46075-2 (PMC10620211; doi:10.1038/s41598-023-46075-2)
Supplement: Supplementary file 7 — Supplementary Table 5. [file 41598_2023_46075_MOESM7_ESM.docx]

Supplementary Table 5 Comparison of R2 and RMSE Values for Patients and Healthy Controls Across SIGH Dimensions

| Population | SIGHD-IDSC Dimensions | Marginal R^2^ Mean | Mean RMSE |
| --- | --- | --- | --- |
| Patients only | SIGH-D | 0.64 | 6.54 |
|  | IDS-C | 0.80 | 19.07 |
|  | SIGH-D IDSC | 0.75 | 16.67 |
| Healthy Controls only | SIGH-D | 0.65 | 5.62 |
|  | IDS-C | 0.71 | 6.53 |
|  | SIGH-D IDSC | 0.70 | 7.13 |
