# Supplementary material for: A smartphone- and wearable-based biomarker for the estimation of unipolar depression severity
Source: Sci Rep. 2023 Nov 1;13:18844. doi: 10.1038/s41598-023-46075-2 (PMC10620211; doi:10.1038/s41598-023-46075-2)
Supplement: Supplementary file 8 — Supplementary Table 6. [file 41598_2023_46075_MOESM8_ESM.docx]

Supplementary Table 6 Average and standard deviation of weekly change in SIGHD-IDSC global and symptom dimensions scores

| SIGH-IDS Categories | Average change in weekly scores (standard deviation) | Average change in weekly scores among MDD  (standard deviation) | Average change in weekly scores among Controls  (standard deviation) |
| --- | --- | --- | --- |
| SIGH-D | -0.68 (±4.13) | -1.02 (±5.63) | -0.33 (±1.38) |
| IDS-C | -0.75 (±6.10) | -0.81 (±8.25) | -0.69 (±2.42) |
| SIGH-IDSC | -1.43 (±9.39) | -1.82 (±12.8) | -1.02 (±3.35) |
| Agitation | -0.02 (±1.32) | -0.02 (±1.80) | -0.02 (±0.49) |
| Anxiety (Psychic) | -0.13 (±1.95) | -0.16 (±2.65) | -0.11 (±0.71) |
| Anxiety (Somatic) | -0.23 (±1.64) | -0.35 (±2.26) | -0.11 (±0.46) |
| Gulit | -0.21 (±1.35) | -0.37 (±1.87) | -0.04 (±0.27) |
| Hypochrondria | -0.04 (±0.45) | -0.05 (±0.61) | -0.04 (±0.19) |
| Interpersonal | -0.04 (±0.74) | -0.11 (±1.01) | 0.02 (±0.23) |
| Mood | -0.27 (±2.87) | -0.37(±3.96) | -0.16 (±0.79) |
| Retard | -0.11 (±0.86) | -0.21 (±1.35) | 0 (±0) |
| Sex | 0.02 (±1.28) | 0.04 (±1.80) | 0 (±0) |
| Sleep | -0.13 (±2.35) | 0.12 (±2.82) | -0.4 (±1.71) |
| Somatic (Gastrointestinal) | -0.06 (±1.25) | -0.05 (±1.68) | -0.07 (±0.50) |
| Somatic (General) | -0.06 (±1.76) | -0.07 (±2.31) | -0.05 (±0.91) |
| Suicide | -0.08 (±0.63) | -0.16 (±0.88) | 0 (±0) |
| Weight | -0.04 (±0.91) | -0.02 (±1.08) | -0.05 (±0.7) |
| Work | -0.21 (±2.00) | -0.28 (±2.74) | -0.13 (±0.61) |
